# O009: “The Chennai declaration”- a historic document (for the high impact paper session)

**DOI:** 10.1186/2047-2994-2-S1-O9

**Published:** 2013-06-20

**Authors:** A Ghafur

**Affiliations:** 1 Apollo Speciality Hospitals, Chennai, India

## Introduction

There is currently no functioning national antibiotic policy or a national policy to contain antimicrobial resistance in India.

## Objectives

“A Roadmap to Tackle the Challenge of Antimicrobial Resistance –the first ever joint meeting of Medical Societies and other stakeholders in India” was held at Chennai on 24th August 2012. The consensus of this historic event was “The Chennai Declaration” which was published in the Indian Journal of Cancer in December 2012.

## Methods

1. The “Roadmap symposium”, per se received extensive coverage in news papers and magazines like “Nature”.

2. Within 2 months of publication of the “Declaration” reputed journal like CID, Lancet ID, JAMA and BMJ published reviews and editorials.

3. Reviews by many other international and Indian journals will be published in the coming months.

4. Popular newspapers in India wrote about the article (“The Hindu” wrote reviews on the document “thrice”).

5. This document is already submitted to the Government and is currently being studied by the highest officials. As a result of the Chennai declaration Ministry of Health (MOH) issued an advertisement regarding antibiotic usage in many Indian news papers. This is only a beginning of a series of changes.

6. The document has also caught serious attention of high level political circles.

7. The article is already accessed by more than 1400 visitors till date from the parent journal website in which it was published. This is in addition to more than 5000 viewers through the ISCCM news letter and also by more than 10,000 physicians in the country, through Association of physicians of India conference update.

## Results

1. The article, within 2 and a half month of publication has literally created a stir among the Indian and international scientific media, public media and the authorities.

2. The paper has in fact changed the attitude of the Indian government towards resistance issue.

3. The declaration has provided an opportunity to the international community to view the problem of antimicrobial resistance in developing countries in a different perspective-A “practical and not a perfect policy” being the ideal approach.

## Conclusion

The Chennai Declaration is a historic document and will be the driving force behind an antibiotic policy in India and other developing countries.

## Disclosure of interest

None declared.
